# Comparison of the Distribution Patterns of Microsatellites Across the Genomes of Reptiles

**DOI:** 10.1002/ece3.70458

**Published:** 2024-11-03

**Authors:** Huaming Zhong, Xuan Shao, Jing Cao, Jie Huang, Jing Wang, Nuo Yang, Baodong Yuan

**Affiliations:** ^1^ College of Biology and Food Shangqiu Normal University Shangqiu Henan China; ^2^ Key Laboratory on Agricultural Microorganism Resources Development of Shangqiu Science and Technology Bureau of Shangqiu City Shangqiu Henan China; ^3^ College of Life Science Liaocheng University Liaocheng Shandong China

**Keywords:** functional annotation, genome, microsatellite, reptiles

## Abstract

Microsatellites or simple sequence repeats (SSRs) are prevalent across various organisms' genomes. However, their distribution patterns and evolutionary dynamics in reptile genomes are rarely studied systematically. We herein conducted a comprehensive analysis of SSRs in the genomes of 36 reptile species. Our findings revealed that the total number of SSRs ranged from 1,840,965 to 7,664,452, accounting for 2.16%–8.19% of the genomes analyzed. The relative density ranged from 21,567.82 to 81,889.41 bp per megabase (Mbp). The abundance of different SSR categories followed the pattern of imperfect SSR (I‐SSR) > perfect SSR (P‐SSR) > compound SSR (C‐SSR). A significant positive correlation was observed between the number of SSRs and genome size (*p* = 0.0034), whereas SSR frequency (*p* = 0.013) or density (*p* = 0.0099) showed a negative correlation with genome size. Furthermore, no correlation was found between SSR length and genome size. Mononucleotide repeats were the most common P‐SSRs in crocodilians and turtles, whereas mononucleotides, trinucleotides, or tetranucleotides were the most common P‐SSRs in snakes, lizards, and tuatara. P‐SSRs of varying motif sizes showed nonrandom distribution across different genic regions, with AT‐rich repeats being predominant. The genomic SSR content of the squamate lineage ranked the highest in abundance and variability, whereas crocodilians and turtles showed a slowly evolving and reduced microsatellite landscape. Gene ontology enrichment and Kyoto Encyclopedia of Genes and Genomes pathway analyses indicated that genes harboring P‐SSRs in the coding DNA sequence regions were primarily involved in the regulation of transcription and translation processes. The SSR dataset generated in this study provides potential candidates for functional analysis and calls for broader‐scale analyses across the evolutionary spectrum.

## Graphical abstract

The distribution patterns and evolutionary dynamics of SSRs in reptile genomes.

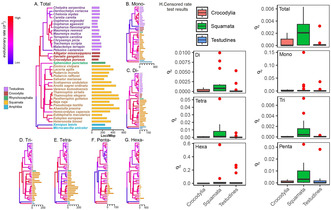

## 1 Introduction

Microsatellites, or simple sequence repeats (SSRs), are tandem repeats of 1–6 bp DNA motifs (Dirlewanger et al. 2004). These noncoding elements are abundant in eukaryotic genomes, spanning intergenic, intron, and coding regions (Pérez‐Jiménez et al. 2013; Phumichai, Phumichai, and Wongkaew 2014). SSRs in coding regions constitute 1%–17% of total SSRs in vertebrate genomes (Varshney et al. 2002) and 0.2%–21% in arthropod genomes (Behura et al. 2011), significantly lower than in noncoding regions. Due to their high polymorphism (Mason 2015), SSRs serve as informative, codominant, multiallele genetic markers. They are extensively used in population genetic structure analysis (Atkinson et al. 2018; Yang, Zheng, and Jin 2019), genetic mapping studies (Leal 2003; Lyons 2001; Ting et al. 2014), genetic relationship identification (Hu et al. 2014; Rayamajhi and Sharma 2018), evolutionary process studies (E et al. 2020; Zhu et al. 2021), and tumor diagnosis (Kloor, Doeberitz, and Gebert 2005; Starostik and Müller‐Hermelink 2001).

Traditionally deemed nonfunctional due to their high instability, SSRs have been found to play key roles in numerous biological processes. They are involved in gene expression, gene silencing, transcription and translation regulation, chromatin organization, genome size regulation, and cell cycle control (Gao et al. 2013; King 2012; Li et al. 2004; Vieira et al. 2016). The functional impact of SSRs on genetic variation can lead to phenotypic changes and the emergence of undesirable traits. Studies on viruses have demonstrated that variations in SSR sequences can modulate the expression of key virulence factors, enhancing their adaptability to diverse environments (Field and Wills 1996). In *Escherichia coli*, high‐frequency mononucleotide and trinucleotide repeats in stress response genes can increase the mismatch rate during replication and transcription, promoting phenotypic diversity (Rocha, Ivan, and François 2002). Microsatellite instability (MSI) within functional genes is associated with various human diseases, including cancers (Hause et al. 2016; Yang, Zheng, and Jin 2019) and neurological disorders (Brouwer, Willemsen, and Oostra 2009; Rohilla and Gagnon 2017).

Examining SSRs at the genomic level is key to understanding organism genomes. Comparing SSRs across taxonomic levels can shed light on the evolutionary importance of these repetitive elements. SSR distribution patterns have been studied in various phylogenetic lineages, including viruses (Zhao et al. 2012), fungi (Karaoglu, Lee, and Meyer 2005), plants (Gao et al. 2013), insects (Ding et al. 2017; Song et al. 2020), fishes (Lei et al. 2021), birds (Fan and Guo 2018), and Euarchontoglire mammals (Song et al. 2021). Reptiles, crucial to both aquatic and terrestrial ecosystems, are vulnerable to habitat degradation and environmental threats. While some research has explored SSRs in specific reptile species (Liu et al. 2019; Pasquesi et al. 2018; Schield et al. 2019; Wei et al. 2020), comprehensive studies on SSR evolutionary dynamics across reptilian taxa are lacking. Conducting in‐depth analyses of reptile SSRs can enhance our understanding of the genomic landscape and evolutionary processes within this diverse group. Future studies could reveal the role of SSRs in reptile evolution and aid conservation efforts for these vulnerable species.

In this study, we identified SSRs across 36 reptile species to clarify the distribution pattern of SSRs in reptilian lineages. Our study revealed divergent SSR composition patterns among squamates, crocodilians, and turtles. The genomic SSR content of the squamate lineage ranks highest in abundance and variability, while crocodilians and turtles display a slowly evolving and reduced microsatellite landscape. We demonstrated that the presence of P‐SSRs in CDSs across reptile genomes showed significant enrichment for transcription‐related functions. This detailed examination of SSR distribution patterns in reptile species provides insights into the biological significance of SSRs in this evolutionarily important lineage.

## 2 Materials and Methods

### 2.1 Data Collection

Publicly accessible genomes and annotation files were downloaded from GenBank (https://www.ncbi.nlm.nih.gov/genome/) and Ensembl (http://ensembl.org/). Subsequently, high‐quality (N50 ≥ 10 Mb) annotated genomes from 36 reptilian species, including 18 serpents, one tuatara, three alligators/crocodiles, and 14 turtles were obtained. These species represent all extant orders of reptiles: Squamata, Rhynchocephalia, Crocodylia, and Testudines. Detailed taxon information, genome size, and accession numbers for the genomes are summarized in Table S1.

### 2.2 SSR Identification and Genomic Location

Before genome sequence analysis, unknown bases (Ns) were filtered using Krait software (Du, Zhang, et al. 2013) to include only valid sequences. SSRs were then identified and localized using Krait. Subsequently, they were classified into P‐SSRs, I‐SSRs, and C‐SSRs. The minimum repeat thresholds for each SSR type were set as follows: 12 for mononucleotide, seven for dinucleotide, and five for trinucleotide, tetranucleotide, pentanucleotide, and hexanucleotide. Repeats with circular permutations and/or reverse complements of each other were grouped as one type for statistical analysis using Krait as previously described (Xu et al. 2018). The resulting tandem repeats were mapped to genic regions, specifically exons, coding DNA sequences (CDSs), and introns, using Krait software (Du, Zhang, et al. 2013).

### 2.3 SSRs Attributes Calculation

To enable parallel comparisons of SSRs among differently sized genomes, we normalized the SSR numbers using relative abundance and relative density with Krait software (Du, Zhang, et al. 2013). Relative abundance was calculated as the number of SSRs per Mb of the total valid length assessed, while relative density was determined by the length of SSRs per Mb of the total valid length analyzed (Du, Li, et al. 2013; Karaoglu, Lee, and Meyer 2005). The correlation between the numbers, length, relative abundance, relative density of SSRs, and genome size was explored using the Pearson correlation coefficient and significance test with R v.4.4.1(R Core Team 2021).

To underscore class‐specific repeat enrichment trends across various taxonomic groups, a heatmap was created, showcasing the density of all P‐SSR motif classes per species. We implemented a scoring system based on Song et al.'s (2021) method with several score modifications. Initially, all repeat motif classes in each genome were ranked by their density. Then, repeats ranked in the top 5, 20, and 35 in the genome received scores of 3, 2, and 1, respectively. The remaining repeats with a density of > 1 were scored to 0. Repeats with a density between 0 and 1 received a score of −1, while all other lower‐end repeats were scored −2. Finally, the clustered matrix of all studied species was visualized using ChiPlot (https://www.chiplot.online).

We used TBtools (Chen et al. 2023) to calculate the GC content of mono‐ to hexa‐P‐SSRs, assessing the GC content variation of P‐SSRs across different reptile subgroups. Each genome assembly was individually extracted for mono‐ to hexa‐P‐SSRs based on their genomic coordinates using Fasta Extrat (Chen et al. 2023). The GC content of these mono‐ to hexa‐P‐SSRs was then determined using Fasta Stats (Chen et al. 2023).

### 2.4 Testing for Multiple Rate Evolution in Microsatellite Abundance Across Lineages

We aimed to test how the overall evolutionary rates of change in SSR abundance varied across major clades in the reptile tree. To test this hypothesis and infer shifts in the rates of evolutionary change in SSR abundance, we conducted a penalized likelihood analysis based on a Brownian motion model for continuous trait evolution of genomic microsatellite class content expressed as loci/Mbp (O'Meara et al. 2006). For all tests, we used the topology and branch lengths derived from Timetree (Kumar et al. 2022). The penalized likelihood analysis was performed using the R v.4.4 (R Core Team 2021) package Phytools 2.0 (Revell 2024), which provides unbiased estimates of the evolutionary rate parameter (*σ* ^2^). Initially, we used edge.widthMap function in Phytools to size the thickness of plotted branches in proportion to the SSRs abundance values. Subsequently, the multirate Brownian evolution model multirateBM was employed to test the variable rates of evolutionary change in SSR abundance. The weight assigned to the penalty term of the fitted model was determined using *λ* (*λ* = 1.0). For each clade and test, rate parameter (*σ* ^2^) estimates were computed for each motif size (mononucleotide to hexanucleotide and for the total SSR loci/Mbp).

### 2.5 Functional Annotation of the CDSs Containing P‐SSRs

CDSs encode proteins, making the distribution of SSRs within CDSs vital for gene function expression. In this study, we used EggNOG‐mapper to conduct gene ontology (GO) enrichment and Kyoto Encyclopedia of Genes and Genomes (KEGG) pathway analysis, exploring the potential roles of SSRs in gene function. EggNOG‐mapper, which relies on the eggNOG database (Huerta‐Cepas et al. 2019) of ortholog groups, covering 5090 organisms, enables mapping of proteins, CDS, genomic, and metagenomic data to annotate GO terms and KEGG pathways. Initially, we downloaded the CDS sequences for each genome. We then annotated both GO terms and KEGG pathways using eggNOG‐mapper (Cantalapiedra et al. 2021). The output annotation which includes the IDs of CDS and GO/KEGG, was set as the background file. The IDs of the CDSs containing P‐SSRs were set as the query to perform GO/KEGG enrichment analysis using TBtools (Chen et al. 2023). Finally, we used ChiPlot to draw bar plots illustrating the enrichment results. Due to computational precision limitations, the *p*‐values of certain KEGG pathways in 16 species are approach zero (KEGG results). Consequently, log10 transformation for data preprocessing was not feasible for visualization in these species, so the visual representation of KEGG analysis only included the other 20 species.

## 3 Results

### 3.1 Occurrence of SSRs

In the present study, we identified SSRs from the genome data of 36 reptile species. Table S2 provides a summary of the taxonomic classification of each organism and the basic attributes of different SSR categories. The total number of SSRs ranged from 1,840,965 to 7,664,452, with a relative density ranging between 21,567.82 and 81,889.41 bp/Mb. Among all SSR categories, I‐SSRs were the predominant class in terms of length proportion (68.85%–89.15%), followed by P‐SSRs (9.97%–23.05%) and C‐SSRs (0.67%–8.25%, Figure S2). The proportion of total SSR length in each genome ranged from 2.16% to 8.19%. Snake species exhibited the highest proportion of all SSRs in their genomes (5.28%–8.19%, averaging 6.83%), followed by lizards (3.17%–5.98%, averaging 4.51%), and tuatara (4.07%). Notably, crocodilians and turtles showed comparably low coverage of SSRs, ranging from 2.55% to 2.47%.

We used Pearson correlation analysis to investigate the relationship between the number, length, abundance, and density of SSRs and genome size. The results showed a positive correlation between the number of SSRs and genome size (*r* = 0.48, *p* = 0.0034) (Figure S1). However, there was a significant negative relationship between genome size and both the loci/Mbp frequency (*r* = −0.41, *p* = 0.013) and bp/Mbp density (*r* = −0.42, *p* = 0.0099) of SSRs (Table S3). Interestingly, the length of SSRs did not correlate with the genome size (*r* = 0.18, *p* = 0.28). Furthermore, the numbers of SSRs in Squamata (*r* = 0.67, *p* = 0.0023) and Tesudines (*r* = 0.7, *p* = 0.005), as well as the length of SSRs in Tesudines (*r* = 0.69, *p* = 0.0061), exhibited a positive correlation with genome size.

### 3.2 Variation Characteristics of P‐SSRs Across Evolutionary Landscape

**FIGURE 1:**
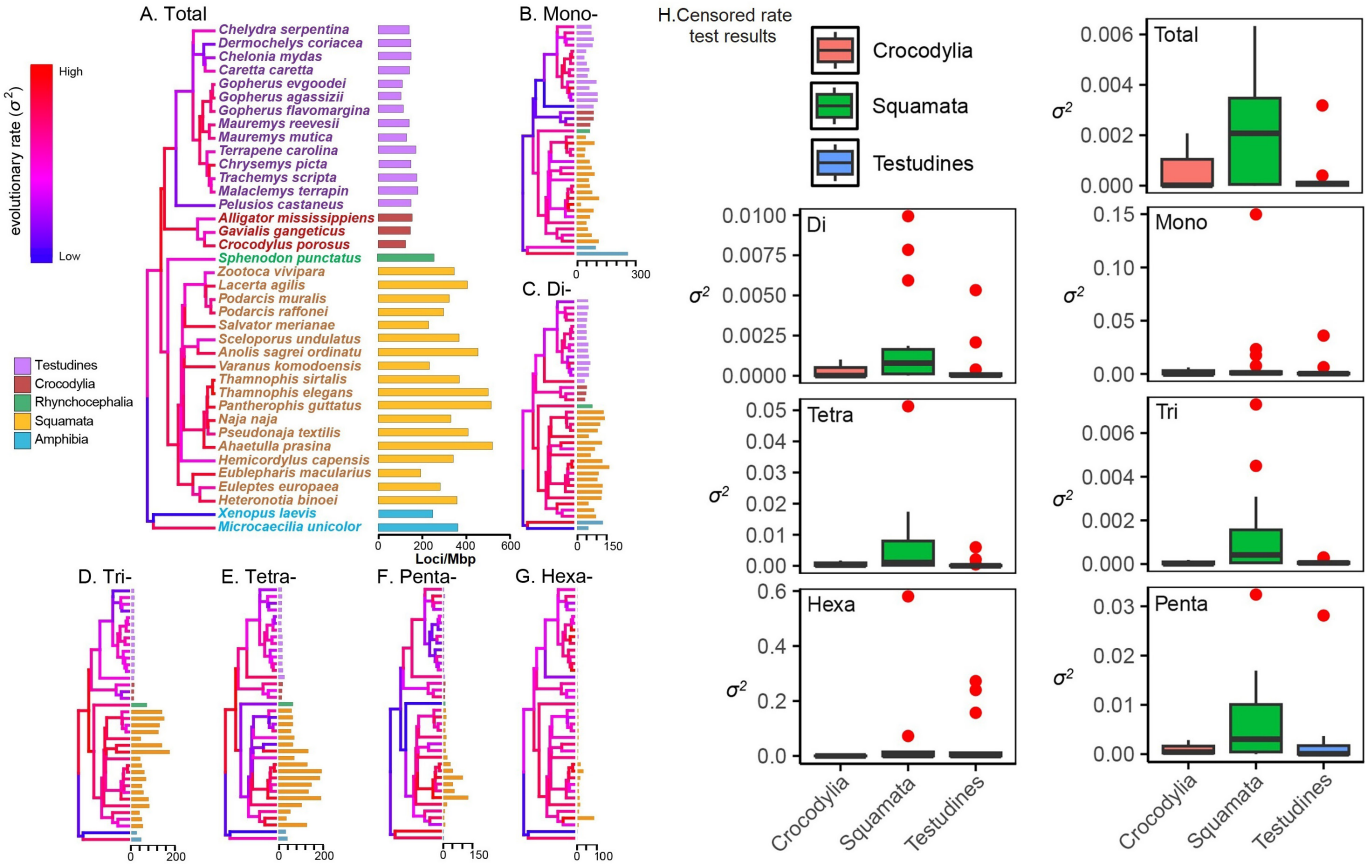
Observed simple sequence repeat (SSR) loci/Mbp frequencies and their lineage‐specific evolutionary rates across 36 reptile taxa. Horizontal bar plots represent the observed SSR loci/Mbp frequencies for each reptile genome. Branches on the time‐calibrated consensus phylogeny are colored according to the estimated rate of microsatellite evolution, with blue indicating slower evolution and red indicating more rapid evolution. Taxa and coinciding bars are consistently colored and ordered based on the four reptile clades. Results are shown for (A) total genomic microsatellite content, (B) Mono‐, (C) Di‐, (D) Tri‐, (E) Tetra‐, (F) Penta‐, (G) Hexa‐ loci/Mbp frequencies, (H) Censored rate test results for lineage‐specific rates of microsatellite evolution across the three major reptile clades. Box pots represent the rate parameter (*σ* ^2^) estimates. The phylogenetic tree was derived from TimeTree (http://www.timetree.org/).

Squamates exhibit the highest average SSR loci/Mbp frequencies (mean = 393.82 loci/Mbp) and abundance variance (SD = 104.92), followed by *Sphenodon punctatus* (282.56 loci/Mbp; Figure 1A, Table S3). On average, crocodilian and turtle genomes contain similarly low SSR abundances (mean = 155.22 and 156.61 loci/Mbp, respectively) with minimal variance within each clade (SD = 17.08 and 25.54, respectively; Figure 1A). Crocodilians and turtles have similar, higher average densities of mononucleotide loci (means = 79.33 and 72.83 loci/Mbp, respectively; Figure 1B), while squamate and *S. punctatus* genomes exhibit similar loci/Mbp densities (68.02 and 65.9 loci/Mbp). Regarding dinucleotide repeats, squamate genomes contain similarly higher average densities (95.2 loci/Mbp) and abundance variance (SD = 24.92), followed by *S. punctatus* (68.16 loci/Mbp; Figure 1C). On average, crocodilian and turtle genomes contain similarly low total abundances of dinucleotide SSRs (mean = 39.89 and 46.92 loci/Mbp, respectively) with minimal variance within each clade (SD = 3.94 and 6.27, respectively; Figure 1C). Similar to trends in loci/Mbp frequencies of dinucleotide repeats, tri‐, tetra‐, penta‐, and hexa‐ repeats also exhibit high average abundance and variance in squamates, while crocodilians and turtles have similar, lower average loci/Mbp densities (Figure 1D–G). We observed clade‐specific rates of evolution in SSR genomic density. Total SSR loci/Mbp abundances evolved rapidly in squamates (means *σ* ^2^ = 0.00225), while crocodilians and turtles exhibited lower rates of change (means *σ* ^2^ = 0.000699 and 0.000311, respectively; Figure 1A,H). For mononucleotide to hexanucleotide SSRs, rates of change in SSR abundance followed the same pattern, with the highest in squamates, followed by turtles and crocodilians (Figure 1B–H).

**FIGURE 2:**
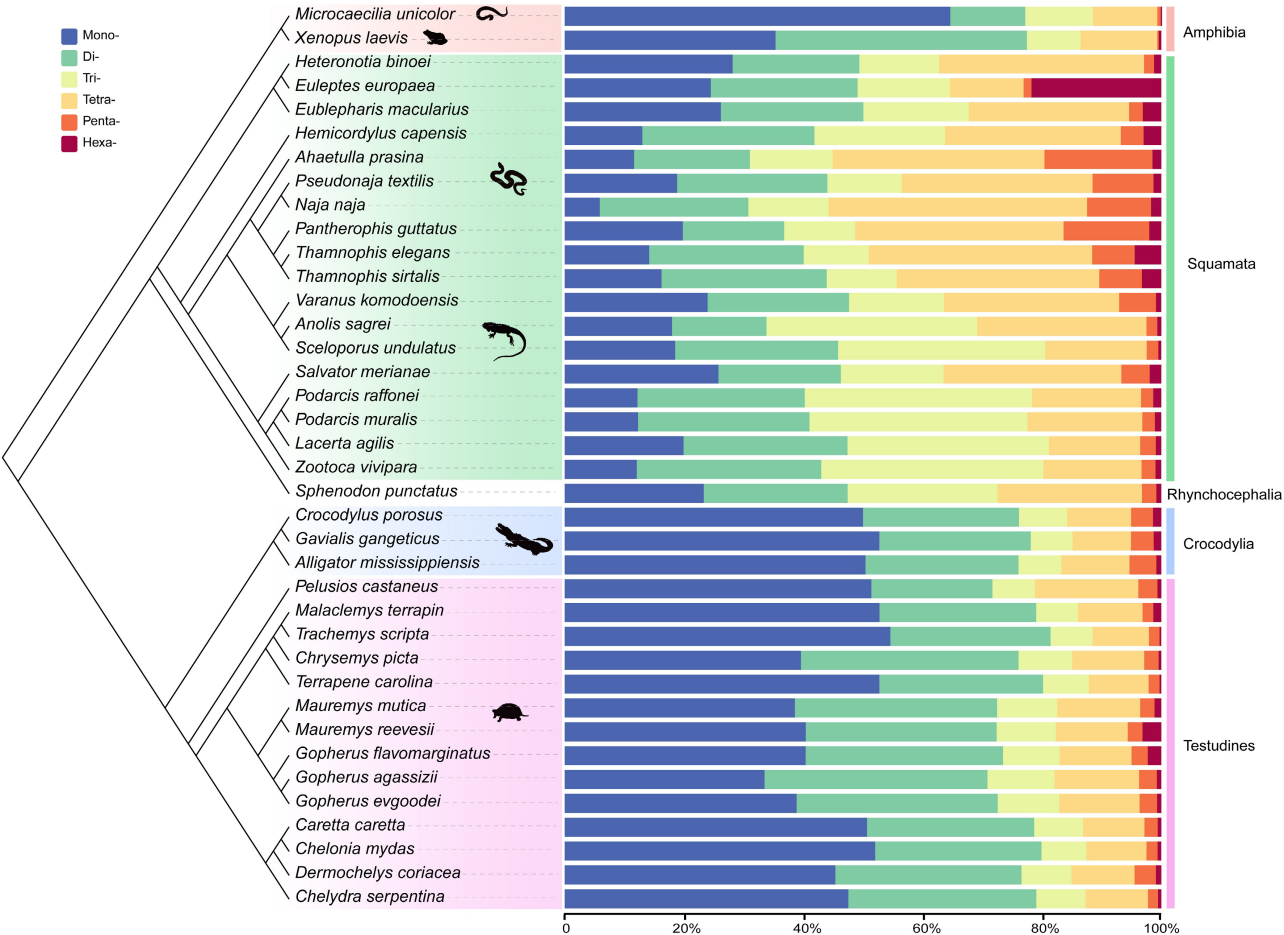
Percentage of six categories of perfect simple sequence repeats (P‐SSRs) in the 36 reptile genomes. Percentages were calculated based on the total number of each P‐SSR type divided by the total number of P‐SSRs in that species. The phylogenetic relationship and divergence times of the 36 species and two outgroups were sourced from TimeTree (http://www.timetree.org/).

We examined the distribution patterns of P‐SSRs across the genomes of reptiles (Figure 2). The dominant P‐SSRs categories in crocodilians and turtles were nearly identical, specifically mononucleotide P‐SSRs. Among snakes, specifically in the Elapidae (2 species) and Colubridae (4 species) families, tetranucleotide P‐SSRs were the most abundant motif (Table S4). In contrast, the most prevalent motif types varied across different lizard genomes. For instance, trinucleotide P‐SSRs were predominant in *Anolis sagrei*, *Sceloporus undulatus* and in the Lacertidae family (four species). *Varanus komodoensis*, *Hemicordylus capensis*, *Salvator merianae*, *Heteronotia binoei*, and *Eublepharis macularius* exhibited tetranucleotide P‐SSRs as the most prevalent motif (Table S4). Notably, in *Euleptes europaea*, dinucleotide P‐SSRs (24.61%) were the most prevalent motif type in *Euleptes europaea*, slightly surpassing mononucleotide P‐SSRs (24.49%).

**FIGURE 3:**
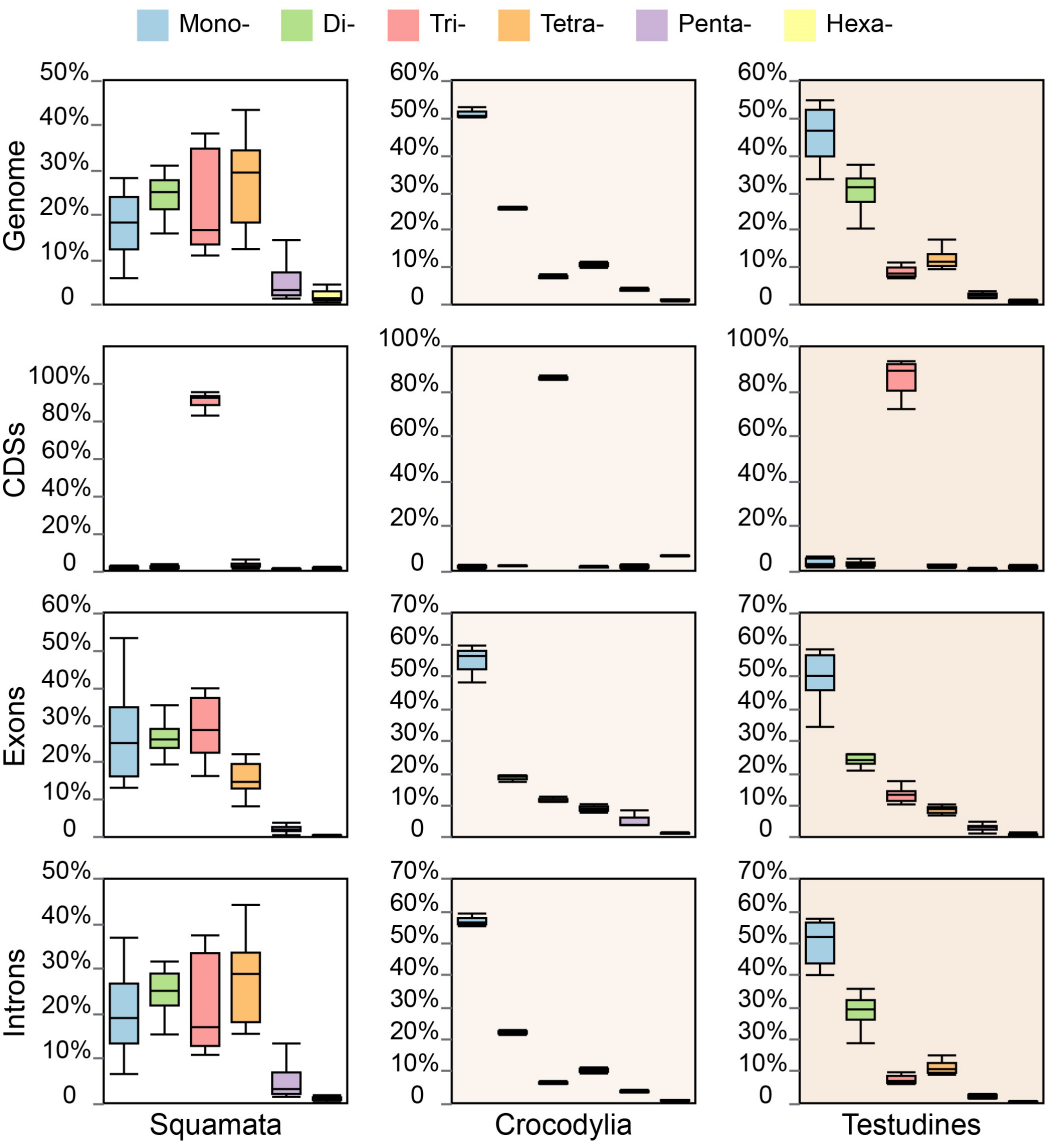
Proportion of mononucleotide to hexanucleotide perfect simple sequence repeats (P‐SSRs) in different genic regions. The tuatara was excluded from the calculation because it is the only surviving species in the Rhynchocephalia order.

We further investigated the abundance of P‐SSRs with varying motif sizes in genic regions and observed taxonomically specific distribution (Figure 3). Our findings indicated a nonrandom distribution of SSR types in genic regions. Trinucleotide P‐SSRs were predominant in the CDSs of all reptile species examined, ranging from 68.6% to 95.49% (Table S4). Conversely, other P‐SSRs classes were infrequent in the CDS regions. Crocodilians and turtles displayed a predominance of mononucleotide repeat motifs in exon regions. In contrast, squamates' exon regions contained abundant mono‐, di‐, or trinucleotide repeat motifs. In the intron region, mononucleotide P‐SSRs were the most common type in crocodilians and turtles. Among squamates, snakes consistently exhibited a dominance of tetranucleotide P‐SSRs in introns, whereas the P‐SSR types varied among lizard species (mono‐, tri‐, and tetranucleotide, Table S4).

**FIGURE 4:**
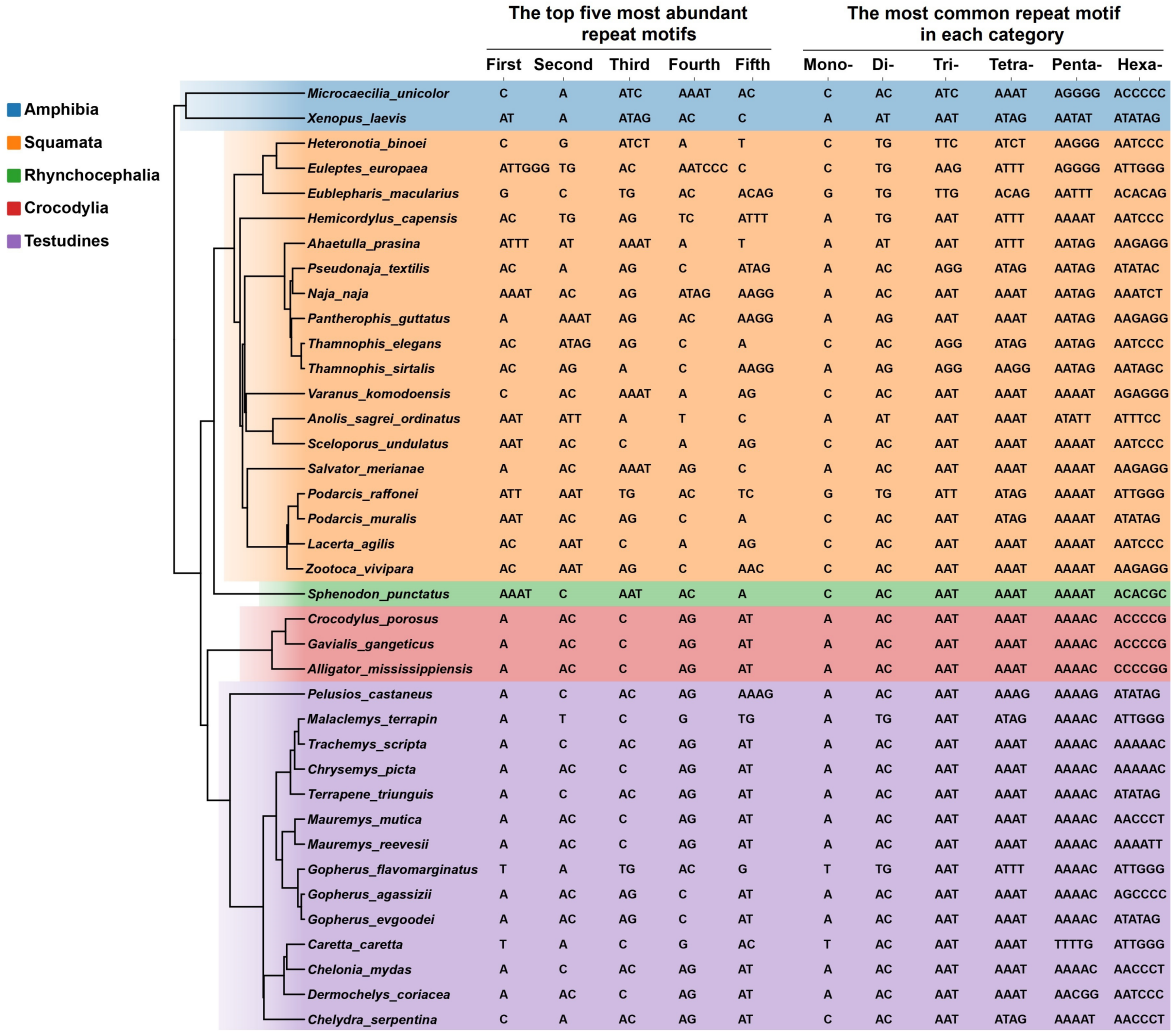
Top five most abundant repeat motifs and the most common repeat motif in each perfect simple sequence repeat (P‐SSR) category in reptile genomes. The phylogenetic tree was derived from TimeTree (http://www.timetree.org/).

Figure 4 showcases the top five most abundant repetitive motifs and the most common repetitive motifs of varying lengths. It highlights that the most dominant P‐SSR motifs exhibit certain taxonomic characteristics. In squamate genomes, the top five most abundant repetitive motifs span from mono‐ to tetranucleotide, except for hexanucleotide in *Euleptes europaea*. In contrast, in crocodilians and turtles, the predominant motifs are primarily mononucleotide (A, T, C) and dinucleotide repeats (AC, AG). Both the dominant motif and the top five motif patterns appear to be conserved in crocodilians and turtles. However, the top five motif patterns show significant variation among squamates. Motifs within each type of SSR were observed to vary somewhat among different genomes. In addition, the results indicated that the pattern of mononucleotide to hexanucleotide repeats was conserved in crocodilians and turtles while exhibiting significant variation among genomes of other orders. 5mer SSRs in snake and lizard genomes appear to be conserved with (AATAG)n and (AAAAT)n, respectively (Figure 4).

We investigated the “top five” and the “most dominant” in different genomic regions (Table S5). Based on previous results, trinucleotide motifs were predominant in the CDSs of nearly all species. We found that trinucleotide motifs, particularly (AGG)s, (ACG)n, (CCG)n, (AAG)n, (ACC)n, and (AGC)n, frequently appeared among the top five motifs in most species. Additionally, the distribution of the most abundant mono‐ to hexanucleotide P‐SSRs varied across different genic regions. As expected, the distribution pattern in intron regions mirrored that in the genomes. However, the dominant P‐SSR repeat motifs in CDSs and exons showed some variations compared to other regions. For example, in the CDSs of crocodilians and turtles, the most abundant mono‐, di‐, and trinucleotide motifs were (C)n, (AG)n, and (AGG)n, respectively, contrasting with (A)n, (AC)n, and (ATT)n in other regions (Table S5).

**FIGURE 5:**
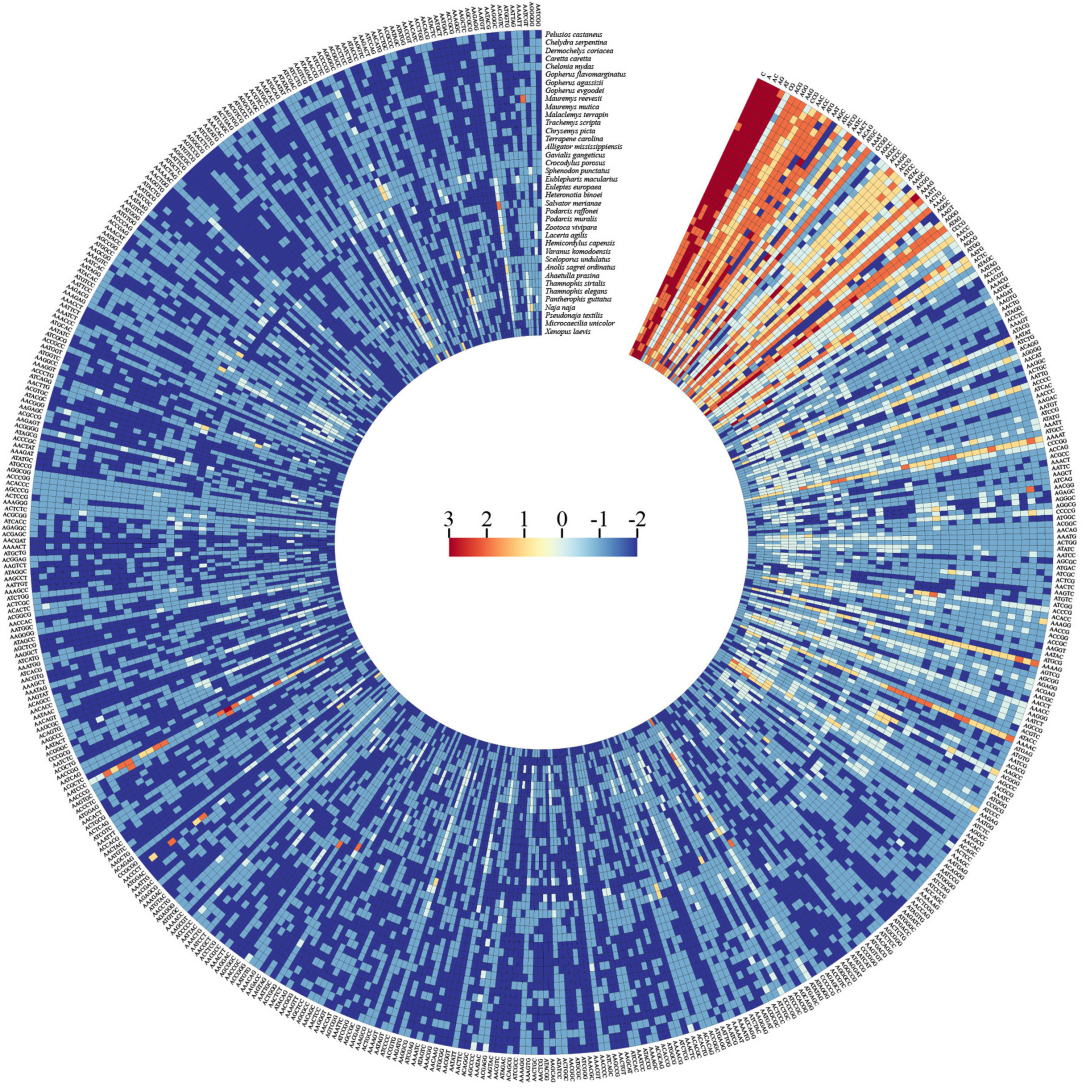
Enrichment trend of perfect simple sequence repeats (P‐SSRs) across reptile genomes. A heat map was generated based on P‐SSR density‐based ranking per the indicated color scale. The P‐SSR motifs are arranged in columns, and the organisms are arranged in rows.

We created a heat map with ranked P‐SSR density to depict the abundance trends of the 501 SSRs across all studied genomes (Figure 5 and Table S6). The heat map unveiled unique abundance patterns among different subgroups. Some P‐SSR motifs, such as (C)n, (AAAG)n, (A)n, (AC)n, and (AG)n, were highly abundant in the majority of organisms. Moreover, certain motif types displayed higher density in specific groups while being relatively scarce in others. For example, the density of (ACTG)n and (ATGAG)n was significantly higher in snakes compared to other clades.

### 3.3 GC Content of P‐SSRs

**FIGURE 6:**
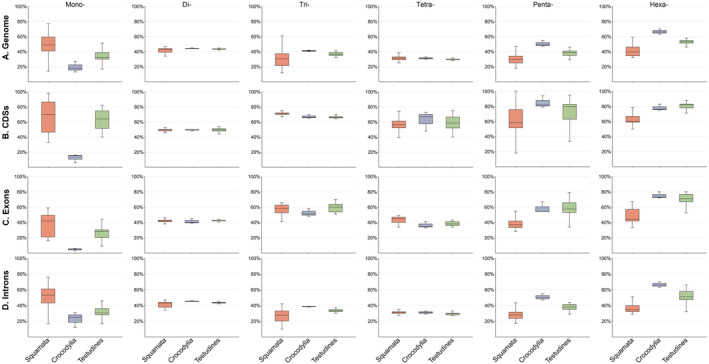
The Guanine–cytosine (GC) content of perfect simple sequence repeats (P‐SSRs) in the genomes and genomic regions of different taxon clades within reptiles. (A) Genomes; (B) CDSs; (C) exons; (D) introns. The tuatara was excluded from the calculation due to it being the only surviving species in the Rhynchocephalia order.

We analyzed GC content variation of P‐SSRs across various reptile orders (Table S7). Figure 6 illustrates the GC content in relation to the genome and genic regions (CDSs, exons, and introns). Predominantly, AT‐rich repeats predominated were observed. Hexanucleotide P‐SSRs had the highest average GC content across all studied genomes, while tetranucleotide P‐SSRs had the lowest. In genic regions, the average GC content of each P‐SSR repeat type in CDS and exon regions exceeded that in genomes and introns. The lowest average GC content was seen in tetranucleotide P‐SSRs, mirroring that in genomes (Figure 6B–D). The repeat type with the most GC content variation differed among genomic or genic regions (Table S7). At the genomic level, mononucleotide P‐SSRs showed the most GC content variation among all species studied, ranging from 13% (*Crocodylus porosus*) to 77% (*Anolis sagrei*). However, no taxon‐specific features were observed in reptiles concerning the GC content of different P‐SSR categories.

### 3.4 Functional Analysis of CDSs With P‐SSRs

**FIGURE 7:**
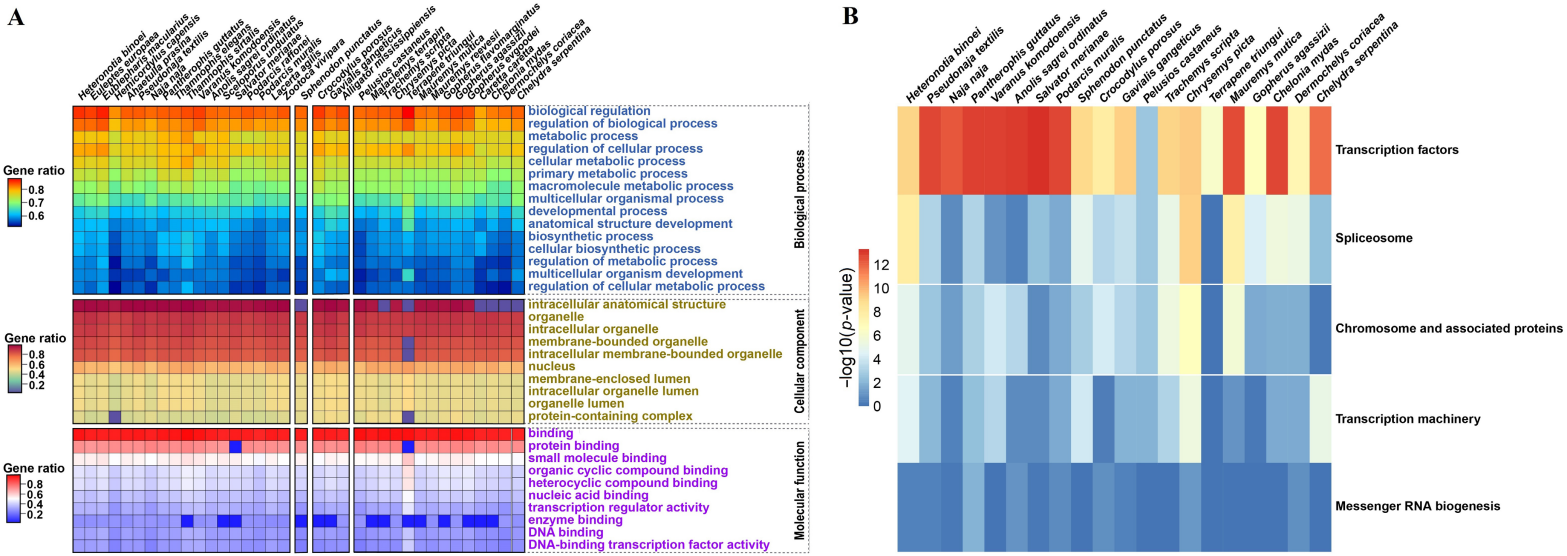
Gene ontology (GO) function annotation (A) and Kyoto Encyclopedia of Genes and Genomes (KEGG) enrichment pathway classification (B) of coding DNA sequences (CDSs) with perfect simple sequence repeats (P‐SSRs) in the genomes of reptiles.

To explore the potential function of genes with P‐SSRs in CDS regions, we conducted a GO enrichment analysis across 36 reptile species. Table S8 encapsulates this information. Figure 7A depicts the gene ratio of CDSs with P‐SSRs assigned to GO categories. Among the top 10 CC categories, the most common classes were “intracellular anatomical structure,” “organelle,” and “nucleus”. The MF category showed significant enrichment in “binding,” and “transcription regulator activity” functions. Genes with P‐SSRs in the CDSs demonstrated numerous associations with BP, including terms like “biological regulation,” “metabolic process,” and “multicellular organism development.” We conducted a KEGG pathway analysis to clarify the biological pathways and functions of CDSs with P‐SSRs. Detailed results are summarized in Table S9. In *Anolis sagrei*, for instance, 1545 genes were allocated to 23 KEGG pathways. The findings indicated that the enriched target CDSs across all genomes were primarily associated with “transcription factors” (Figure 7B). The most abundant transcription factors containing P‐SSRs shared among different reptile clades were SRY‐box transcription factor (SOX) and forkhead‐box protein (FOX, Table S10).

## 4 Discussion

### 4.1 The Distribution Patterns of SSRs

In this study, we identified and compared SSRs from 36 genomes across major reptilian lineages. Our results suggest that snake genomes are relatively enriched in SSRs, while crocodilian and turtle genomes exhibit a reduced presence of SSRs. This finding is consistent with previous studies (Adams et al. 2016; Castoe, de Koning, et al. 2011; Castoe, Hall, et al. 2011; Pasquesi et al. 2018; Shedlock et al. 2007). The proliferation of SSRs in snakes suggests a potential association between SSR expansion and the adaptive evolution of snakes. Generally, the SSR coverage in most reptile genomes (2.55%–8.19%) surpasses that in insect genomes (0.02%–3.1%) (Ding et al. 2017), but is marginally lower compared to Euarchontoglires genomes (3.19%–9.87%) (Song et al. 2021).

Prior research has established a strong correlation between the quantity of repetitive DNA and genome size in eukaryotes (Bennetzen, Ma, and Devos 2005; Blommaert et al. 2019). Our study corroborates this, revealing a positive correlation between the count of SSRs and genome size. However, we found an inverse relationship between frequency and density of SSRs genome size across all examined organisms. We also observed varying correlations between genome size and SSR characteristics across different taxonomic subgroups. For instance, in squamates, the number of SSRs positively correlated with genome size. Similarly, in turtles, both the total count and length of SSRs showed a positive correlation with genome size. This correlation is consistent with findings in viruses (Zhao et al. 2012), insects (Ding et al. 2017), and Euarchontoglires (Qi et al. 2016; Song et al. 2021), suggesting a universal relationship between SSR count (or length) and genome size across organisms. However, the correlation between genome size and SSR density (or abundance) appears to be clade‐specific. We noted a significant negative correlation between SSR abundance (or density) and genome size. This is in line with observations in fungi (Wang et al. 2014), bovid species (Qi et al. 2015), and plants (Morgante, Hanafey, and Powell 2002), where SSR abundance exhibited a negative correlation with genome size. Conversely, recent studies on insects (Ding et al. 2017), birds (Huang et al. 2016), and rodents (Song et al. 2021) found no correlation or significant relationship between SSR abundance and genome size. Thus, the relationship between genome size and SSR density (or abundance) may vary among different clades.

The abundance of SSRs differs across various genic regions. Typically, the diversity of SSR loci in coding regions is significantly less than in noncoding regions. This is likely due to the high polymorphism of SSR loci, which can lead to frameshift mutations in coding regions, resulting in significant changes in amino acid sequences that may be removed by negative natural selection (Metzgar, Bytof, and Wills 2000). The reduced diversity of SSRs in coding sequences compared to noncoding regions also suggests that coding sequences are subject to greater selective pressure.

### 4.2 Taxon‐Specific Features of P‐SSRs

While SSR distribution patterns have been extensively studied in a broad spectrum of taxonomically diverse organisms, our understanding of SSR characteristics in reptiles across various taxonomic levels remains limited. Our study found that the most abundant P‐SSR category was conserved (mononucleotide) in crocodilians and turtles. In contrast, squamates displayed significant variations in the most common P‐SSRs, ranging from mononucleotide, trinucleotide, to tetranucleotide motifs. This distribution pattern not only surpasses the diversity observed in other reptilian clades, but also exceeds the diversity seen in other animal species. A previous study also reported a similar extraordinary abundance of microsatellites in squamate genomes (Pasquesi et al. 2018). For example, in primates, tree shrews, and colugo genomes, the mononucleotide P‐SSR category was found to be the most abundant. Rodents and lagomorphs demonstrated the highest abundance of mono‐ or dinucleotide P‐SSRs (Song et al. 2021). In insect genomes, di‐ and trinucleotides were observed to have the highest average SSR ratios (Ding et al. 2017).

We analyzed the distribution of mono‐ to tetranucleotide microsatellites across various genic regions. We found that mononucleotide P‐SSRs are primarily located in the exons and introns of crocodilians and turtles. However, squamates display significant variation in the dominant repeat motif between exons and introns. Exons mainly contain mono‐, di‐, or trinucleotide SSRs, while introns are characterized by mono‐, tri‐, or tetranucleotide SSRs. Given these significant variations in the most common P‐SSRs, it is plausible that the evolution of SSRs in squamates diverges across different lineages. Additionally, we observed that di‐ and tetranucleotide SSRs are primarily found in noncoding regions. This could be due to the fact that mutations of nontrinucleotide and hexanucleotide SSRs within the coding region can induce frame shifts over time. These shifts can lead to significant changes in protein sequences and potentially give rise to new genes. In contrast, in noncoding regions such as introns or intergenic regions, these repetitive mutations do not result in amino acid changes and have minimal impacts on species (Dokholyan et al. 2000). Consistent with this, CDS regions in the majority of reptile species exhibit a significantly higher proportion of trinucleotide SSRs compared to other regions in the majority of reptile species.

Numerous studies highlight that genomes from different clades display unique repeat motifs. Rodents are characterized by an abundance of (A)n, (AC)n, or (AG)n repeats, while primates predominantly feature (AT)n repeats (Song et al. 2021). The most common repeat motif types in the genomes of four camelid species include (A)n, (C)n, and (AC)n, among others (Manee et al. 2020). We investigated the relationship between SSR distribution patterns and phylogenetic relationships among reptiles. The top five most abundant repeat motifs showed higher conservation levels in crocodilians and turtles compared to squamates. Generally, crocodilians and turtles are characterized by an abundance of short motifs, such as (A)n, (C)n, (AC)n, (AG)n, and (AT)n. In contrast, longer motifs like (AAAT)s, (ATAG)s, and (AAAG)s may also be prevalent in the genomes of other groups. Regarding the most frequent repeat motifs ranging from mononucleotides to hexanucleotides, we observed a taxon‐specific enrichment trend for certain motif types. In line with previous research, we identified (AATAG)n as the most common 5‐mer SSR motif in snake genomes (Adams et al. 2016; Castoe, Hall, et al. 2011; Pasquesi et al. 2018). Pasquesi et al. demonstrated that the largest documented AATAG loci originated from long interspersed nuclear elements. The fluctuating abundance of AATAG loci contributed to the variability in snake genome repeat content on one hand, and on the other, led to a significant increase in AATAG in snake genomes. They established this lineage with the highest genomic SSR content levels among vertebrates (Pasquesi et al. 2018). The prevalence of taxon‐specific repeat units may indicate specific biological functions in certain phylogenetic clades. Research has shown that SSRs located in CDSs can alter protein function and potentially provide a molecular basis for organisms to adapt to changing environments (Kashi and King 2006; Li et al. 2004, 2010; Verstrepen et al. 2005). Within CDSs, (AGG)n, (ACG)n, and (CCG)n emerged as the dominant trinucleotide repeat motifs among the examined genomes. These preferred motifs could undergo repeated transcription in the same amino acids, thereby influencing the physical and chemical properties of the proteins (Saeed, Wang, and Wang 2016).

Variability in GC content serves as a valuable tool for understanding the characteristics of different genomic regions. Prior research has indicated that mononucleotide P‐SSRs in the genomes of beetles (Song et al. 2020), forest musk (Qi et al. 2020), bovids (Qi et al. 2015), and Euarchontoglires have the lowest GC content (Song et al. 2021). This study observed considerable variations in GC content among all species studied for mononucleotide P‐SSRs in genomes, CDSs, and introns. No taxon‐specific features were observed in reptiles regarding the GC content of different P‐SSR categories, suggesting evolving selective constraints on P‐SSR GC content across clades. Furthermore, we noted that the average GC content of each repeat type of P‐SSRs in CDS and exons was higher than that in genomes and introns. Certain GC‐rich SSRs may influence replication by affecting the secondary structure of DNA (Bhati et al. 2010; Nakagama et al. 2006). Therefore, the GC content in SSRs can be considered a critical parameter indicating their functional significance.

### 4.3 Potential Function of CDSs Harboring P‐SSRs

SSRs located in CDSs can modify protein function and contribute positively to the adaptive evolution of organisms in changing environments (Kashi and King 2006; Li et al. 2004, 2010; Verstrepen et al. 2005). Our GO enrichment analysis revealed that CDSs containing P‐SSRs were enriched in functions related to “binding” and “biological regulation” across various reptile species. The KEGG pathway analysis underscored “transcription factors” as the primary function in the CDSs of the representative genomes. Transcription factors play pivotal roles in the transcriptional regulation of organisms through specific DNA sequence binding (Imlay 2015; Symonenko et al. 2018). They likely serve as key regulatory elements governing numerous biological processes, such as ontogenetic development and metabolic processes (Wang et al. 2022; Li et al. 2021; Mejhert et al. 2020). Therefore, it is plausible to speculate that genes containing P‐SSRs may regulate the selective synthesis of specific proteins in reptiles. Our results demonstrated that the forkhead‐box protein and SOX transcription factors were the two most prevalent categories containing P‐SSRs in various reptilian clades. Previous studies revealed that forkhead‐box transcription factors have key roles in various aspects of immune regulation (Coffer and Burgering 2004; Sakaguchi et al. 2010; Zhang et al. 2019). Sox transcription factors are involved in a wide range of developmental processes, such as the regulation of stem cell states, the guidance of differentiation, and the modulation of the local chromatin landscape (Schock and LaBonne 2020). The SOX factors are critical regulators of the male sex determination pathway, particularly in the maintenance of spermatogenesis and adult fertility (She and Yang 2017). Moreover, they are involved in maintaining stem cell pluripotency and promoting differentiation. Specifically, SOX2 is essential for preserving embryonic stem cell pluripotency (Avilion et al. 2003). Moreover, they are associated with a wide range of biological functions and are involved in diverse aspects of growth and development. Overall, the functional annotation results suggest that SSRs within CDSs are predominantly associated with basal metabolism and essential life processes, potentially indicating their role in environmental adaptation.

## Author Contributions

**Huaming Zhong:** conceptualization (lead), methodology (lead), writing – original draft (lead), writing – review and editing (lead). **Xuan Shao:** data curation (equal), methodology (equal). **Jing Cao:** data curation (equal), methodology (equal). **Jie Huang:** data curation (equal), methodology (equal). **Jing Wang:** data curation (equal). **Nuo Yang:** data curation (equal). **Baodong Yuan:** funding acquisition (lead), project administration (lead), supervision (lead).

## Conflicts of Interest

The authors declare no conflicts of interest.

## Supporting information


**Figure S1.** The correlation between the numbers, length, relative abundance, relative density of SSRs, and genome size.

**Figure S2.** Change of evolutionary rate in the abundance of compound SSR (C‐SSRs) and imperfect SSR (I‐SSRs) among reptile species.

**Table S1.** Genome information of 36 reptile and two amphibian (outgroup) species.

**Table S2.** Basic attributes of perfect simple sequence repeats (P‐SSR), compound simple sequence repeats (C‐SSR), and imperfect simple sequence repeats (I‐SSR) in studied genomes.

**Table S3.** Perfect simple sequence repeats (P‐SSRs) loci/Mbp frequencies of mononucleotide to hexanucleotide P‐SSRs in the genome and genic regions.

**Table S4.** Proportion of mononucleotide to hexanucleotide in the genome and genic regions.

**Table S5.** The most abundant perfect simple sequence repeat (P‐SSR) repeat motif in different regions of studied genomes.

**Table S6.** Density of the perfect simple sequence repeat (P‐SSR) motifs across the genome of studied species.

**Table S7.** Guanine–cytosine (GC) content of perfect simple sequence repeats (P‐SSRs) in the genomes and different genic regions of reptile genomes.

**Table S8.** GO enriched in the subset of CDSs containing SSRs in genomes of 36 reptiles. The *p*‐value cutoff is 0.05.

**Table S9.** The KEGG results of the CDSs containing SSRs in genomes of 36 reptiles.

**Table S10.** The transcription factors containing P‐SSRs in genomes of 36 reptiles.

## Data Availability

The genomic locations, motif type, repeat, and gene features of identified SSRs are available at Zenodo (https://doi.org/10.5281/zenodo.10694145).
