# Supplementary figures and images for: Comparison of the Distribution Patterns of Microsatellites Across the Genomes of Reptiles
Source: Ecol Evol. 2024 Nov 3;14(11):e70458. doi: 10.1002/ece3.70458 (PMC11581779; doi:10.1002/ece3.70458)

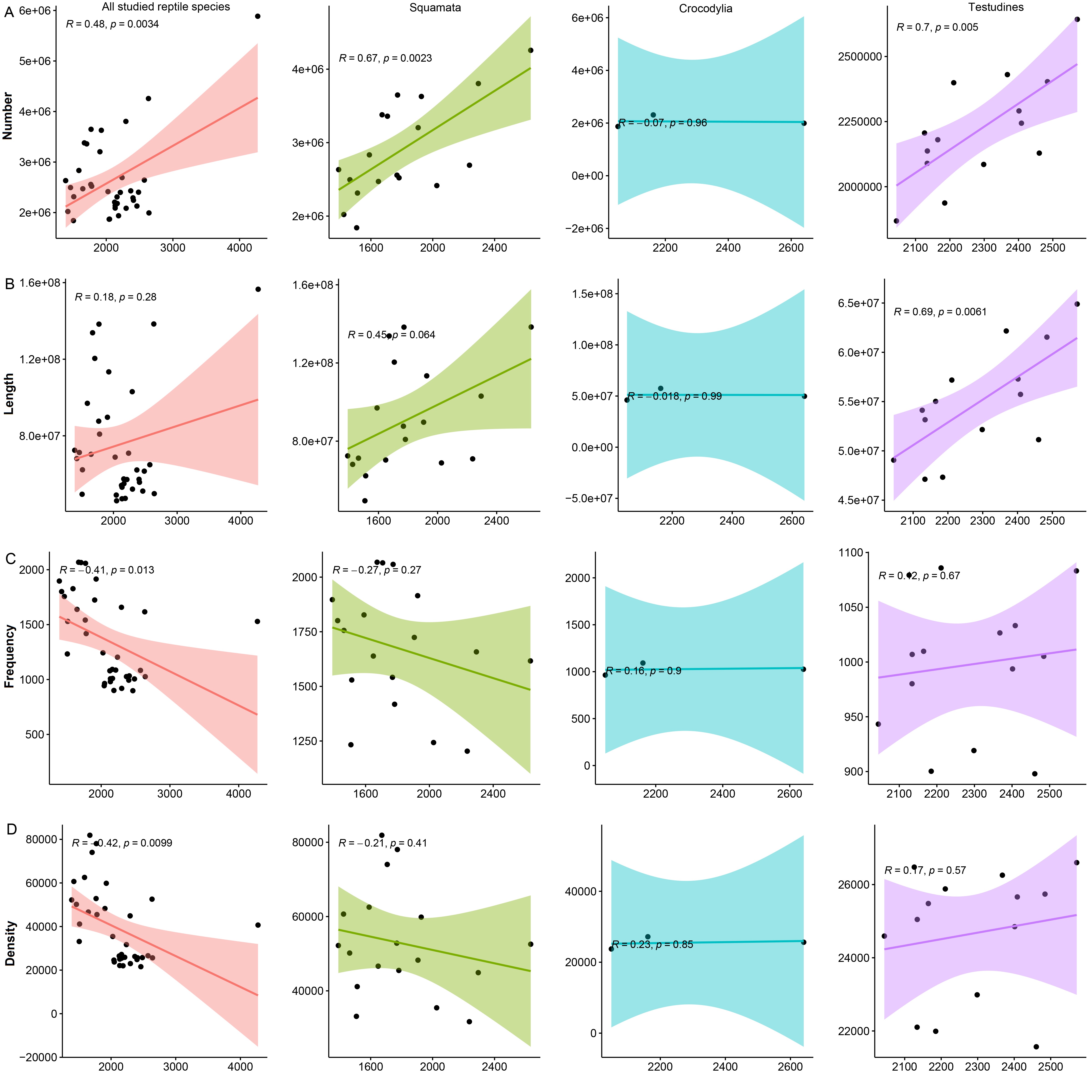

Supplement: Supplementary file 1 — Figure S1. The correlation between the numbers, length, relative abundance, relative density of SSRs, and genome size. [file ECE3-14-e70458-s008.jpg]

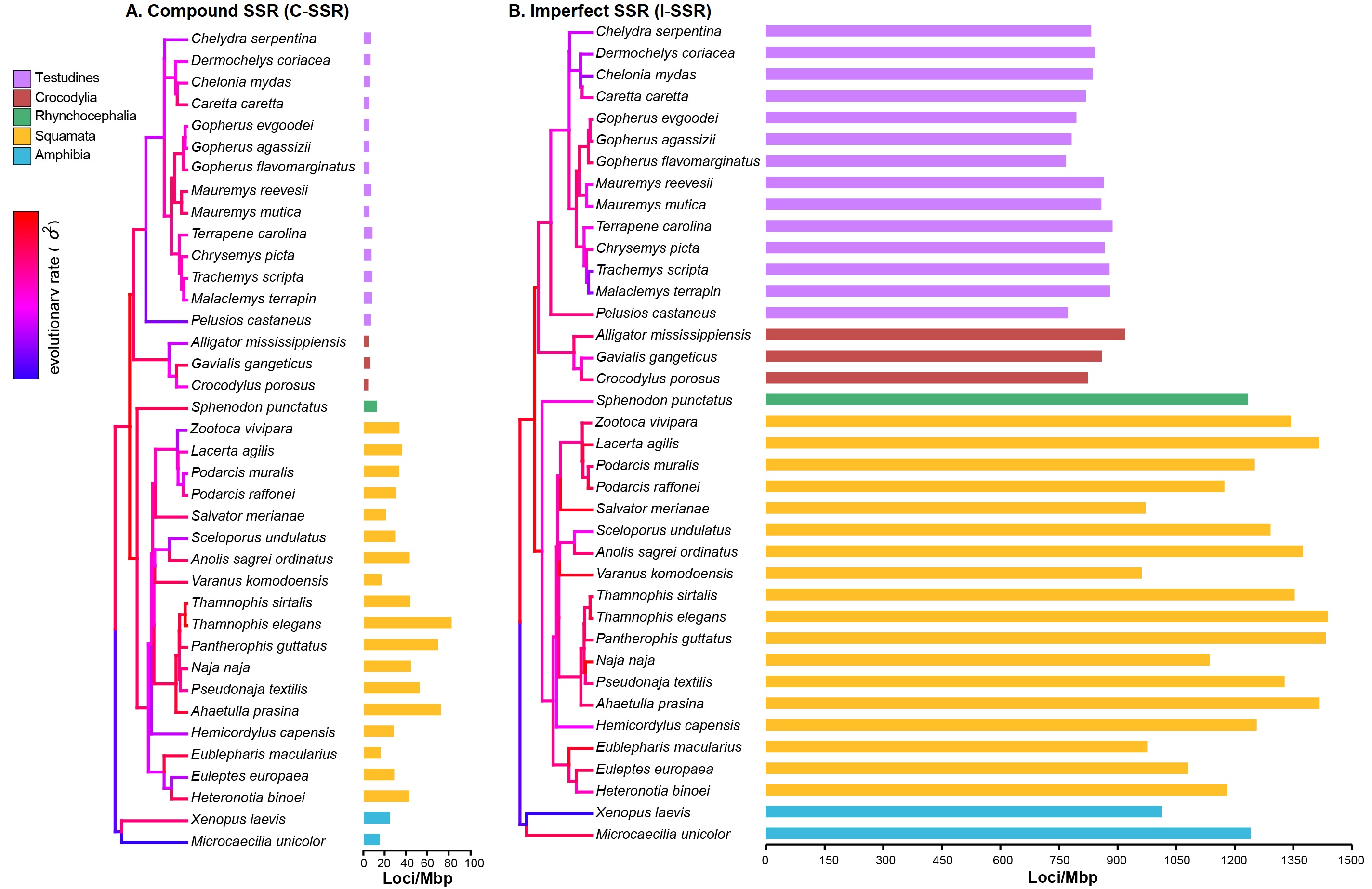

Supplement: Supplementary file 2 — Figure S2. Change of evolutionary rate in the abundance of compound SSR (C‐SSRs) and imperfect SSR (I‐SSRs) among reptile species. [file ECE3-14-e70458-s005.jpg]
